# Thermal Progress of Unsteady Separated Stagnation Point Flow with Magnetic Field and Heat Generation in Hybrid Ferrofluid

**DOI:** 10.3390/nano12183205

**Published:** 2022-09-15

**Authors:** Najiyah Safwa Khashi’ie, Iskandar Waini, Nurul Amira Zainal, Khairum Bin Hamzah, Abdul Rahman Mohd Kasim, Norihan Md Arifin, Ioan Pop

**Affiliations:** 1 Fakulti Teknologi Kejuruteraan Mekanikal dan Pembuatan, Universiti Teknikal Malaysia Melaka, Hang Tuah Jaya, Durian Tunggal 76100, Melaka, Malaysia; 2 Centre for Mathematical Sciences, Universiti Malaysia Pahang, Lebuhraya Tun Razak, Gambang 26300, Pahang, Malaysia; 3 Institute for Mathematical Research, Universiti Putra Malaysia, UPM Serdang, Serdang 43400, Selangor, Malaysia; 4 Department of Mathematics, Faculty of Science, Universiti Putra Malaysia, UPM Serdang, Serdang 43400, Selangor, Malaysia; 5 Department of Mathematics, Babeş-Bolyai University, 400084 Cluj-Napoca, Romania; 6 Academy of Romanian Scientist, Ilfov Street, 050045 Bucharest, Romania

**Keywords:** hybrid ferrofluid, heat generation, magnetic field, separated stagnation point, streamline, unsteady flow

## Abstract

This paper examines the unsteady separated stagnation point (USSP) flow and thermal progress of Fe_3_O_4_–CoFe_2_O_4_/H_2_O on a moving plate subject to the heat generation and MHD effects. The model of the flow includes the boundary layer and energy equations. These equations are then simplified with the aid of similarity variables. The numerical results are generated by the bvp4c function and then presented in graphs and tables. The magnetic and acceleration (strength of the stagnation point flow) parameters are the contributing factors in the augmentation of the skin friction and heat transfer coefficients. However, the enhancement of heat generation parameter up to 10% shows a reduction trend in the thermal rate distribution of Fe_3_O_4_–CoFe_2_O_4_/H_2_O. This finding reveals the effectiveness of heat absorption as compared to the heat generation in the thermal flow process. From the stability analysis, the first solution is the physical solution. The streamline for the first solution acts as a normal stagnation point flow, whereas the second solution splits into two regions, proving the occurrence of reverse flow.

## 1. Introduction

A ferrofluid is a base liquid with colloidal interferences of single-domain ferromagnetic elements, also known as a magnetic colloid. It has a variety of biological and pharmacological applications such as vacuum chambers, revolving shaft seals, radiation dissipation, amplifiers, cell parting, medicine delivery, and computer drives [1]. This type of fluid has distinct properties and responds strongly to magnetization. As a result, it has enormous potential as a new nanotechnology-based heat-transfer fluid with adequate thermal capabilities. Recently, a nanofluid invention known as hybrid nanofluid was initiated. Thus, by combining ferrofluids with this new discovery, hybrid ferrofluids are formed. Hybrid ferrofluids are nanofluids containing several suspensions of nanometer-measured solid ferromagnetic particles in conventional heat exchange fluids which are primarily useful for meeting the needs and demands of manufacturing or innovative firms.

Several studies have been conducted to investigate the efficacy of hybrid ferrofluids as a heat-transfer fluid. Anuar et al. [2] included the magnetic environment in their study of stagnation point flow on exponentially stretching/shrinking surfaces in hybrid ferrofluids. When compared to ferrofluid, they found that hybrid ferrofluids improved the heat transfer rate significantly. Meanwhile, Saranya et al. [3] considered the viscous–ohmic dissipative effect, and it turned out that the Eckert number remarkably reduced both the skin friction coefficient and the heat transfer rate. Waini et al. [4] numerically investigated the unsteadiness parameter in their analysis of hybrid ferrofluid flow. Surprisingly, adding the unsteadiness parameter had a beneficial effect on the thermal rate of their particular study. In another study, Hamid et al. [5] summarized that the kerosene-based ferrofluid demonstrated a higher convective heat transfer rate than the water-based ferrofluid. From the above-stated studies, it is proven that hybrid ferrofluids may help to intensify the heat transfer rate with the addition of other appropriate governing parameters.

The heat generation or absorption consequences may alter the temperature distribution in some applications, such as those dealing with dissociating fluids and chemical reactions. This might eventuate in applications such as electronic chips, semiconductor wafers, and nuclear reactors [6]. Previous research on temperature-dependent heat sources or sinks for various geometries is widely available. Zainal et al. [7] performed a numerical analysis of unsteady stagnation point flow in hybrid nanofluids with a heat generation effect. Elbashbeshy et al. [8] presented that, as the parameter is switched from heat absorption to heat generation, the Nusselt number decreases, and the Sherwood number increases. Khan et al. [9] demonstrated that increasing heat generation and the Eckert number lowers the temperature profile, while increasing the heat absorption parameter shows the opposite effect. As a result, the thickness of the thermal boundary layer increases and decreases for heat generation and heat absorption, respectively. A few more studies on the influence of heat generation/absorption can be found in [10,11,12,13,14,15].

Recently, the topic of unsteady flow through the boundary layer and heat transfer has sparked the interest of researchers. This growing interest is fueled by their numerous practical applications in engineering and industrial processes such as the extraction of polymer and rubber sheets, wire drawing, and glass fiber production. Normally, the ideal flow environment around the system is assumed to be steady, but unpredictable effects of the unsteadiness parameters occur due to fluctuations or nonuniformities or body self-induction in the surrounding fluid. Moreover, some devices must execute time-dependent motion in order to perform their basic functions [16]. According to Waini et al. [17], Wang [18] pioneered the development of the unordinary type of flow caused by shrinking when he explored the behavior of a liquid film on an unsteady stretching sheet. Since then, many researchers have extensively investigated the unsteady flow in various cases, especially in hybrid nanofluids. Bhandari [19] scrutinized the unsteady flow of ferrofluid between two shrinking discs under magnetic field influence and heat transfer. Islam et al. [20] examined the unsteady effect in ferrofluid with the inclusion of convective boundary conditions. Meanwhile, Hussain et al. [21] evaluated the forced convection flow of ferrofluids toward a rotating cylinder utilizing a one-phase nanofluid model by considering the inclined magnetic field. There are numerous additional references for the unsteady flow in hybrid nanofluids in earlier studies [22,23,24,25,26,27,28]. Meanwhile, critical review papers on steady and unsteady flow are available in [29,30].

Another factor that should be considered in the study of hybrid nanofluid flow is the location where the fluid is examined. A popular case is the flow over a stagnation point, which describes the fluid motion near the region of a surface occurring in a fixed or moving body. However, the flow of the stagnation point should be further considered with other surfaces. For instance, Hiemenz [31] studied stagnation point flow toward a stationary semi-infinite point in a two-dimensional region, and the ideas were later extended to an investigation on axisymmetric stagnation point flow by Homann [32]. The study on stagnation point flow was furthered by considering non-Newtonian fluid. For example, Lund et al. [33] proposed the formulation of MHD stagnation point flow involving Casson fluid with thermal radiation and viscous dissipation circumstances. Due to the interest in that particular study, many researchers have made further contributions [33,34,35,36,37,38]. In the literature, both nanofluid and hybrid nanofluid have been considered in the case of stagnation point flow [39,40,41,42].

Motivated by previous studies, with the aim of filling the research gaps, the current study contributes to the analysis of unsteady separated stagnation point flow (USSP) of Fe_3_O_4_–CoFe_2_O_4_/H_2_O with the presence of a heat generation effect. The authors believe that the present findings are valuable within the current research trend of boundary layer flow analysis. Some assumptions for the physical model are applied and mathematically modeled. The governing model is then simplified into similarity (ordinary) differential equations and solved numerically using the bvp4c solver. Furthermore, the impact of physical parameters, such as the magnetic field, heat generation, and acceleration, on the distribution of skin friction coefficient and thermal rate is analyzed. The final analysis results are portrayed in the form of figures and tables. Stability analysis is also conducted to validate the reliability of the physical solution. These novel findings can assist other researchers and scientists in expanding their knowledge of this prospect fluid, especially in heat transfer and boundary layer analysis of USSP flow.

## 2. Mathematical Formulation

**Figure 1:**
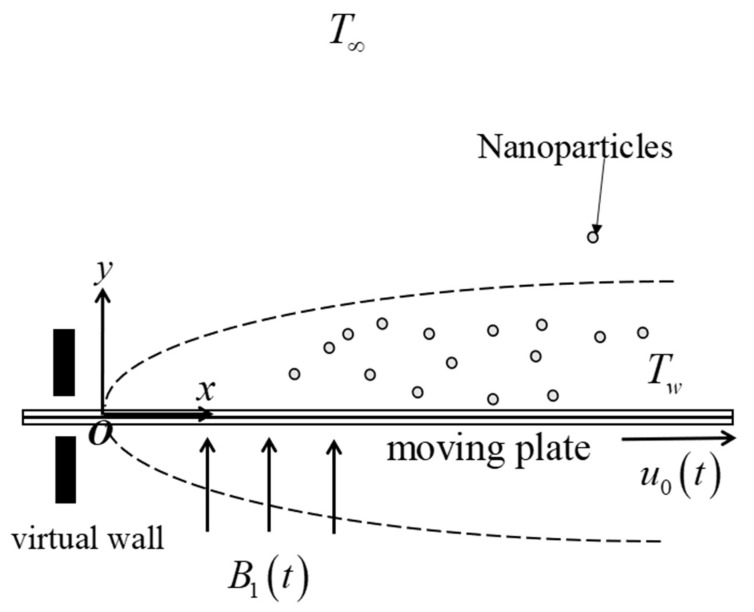
The physical model.

The heat transfer and USSP flow of Fe_3_O_4_–CoFe_2_O_4_ (magnetite–cobalt ferrite) with water-based fluid (H_2_O) toward a moving plate with a magnetic field and heat generation are examined, as illustrated in Figure 1. The following physical assumptions are considered:

- The plate velocity is $u_{0}\left(t\right)=\partial x_{0}\left(t\right)/\partial t$, where t and $x_{0}\left(t\right)$ are the time and plate’s displacement, respectively (see Dholey [43] and Khashi’ie et al. [44]).
- The free stream velocity (parallel to the plate) is $u_{e}\left(x,t\right)=\alpha \frac{\left(x-x_{0}\left(t\right)\right)}{t_{ref}-\beta t}+u_{0}\left(t\right)$, where $t_{ref}$ and t are the constant reference time and time, respectively (see Dholey [43] and Khashi’ie et al. [44]). In addition, α refers to the strength of free stream velocity, denoted as the acceleration parameter. This velocity is also located outside the boundary layer region.
- Importantly, β is an unsteadiness parameter, where β=0 denotes a steady boundary layer flow, while β>0 and β<0 refer to unsteady accelerating and decelerating parameters, respectively.
- For the heat generation effect, $Q_{1}=\frac{Q_{0}\left(x-x_{0}\right)}{\left(t_{ref}-\beta t\right)}$ is the variable heat generation factor, where $Q_{0}$ is a constant (see Kumbhakar and Nandi [28]).
- For the magnetic field effect, $B_{1}=\frac{B_{0}}{\sqrt{\nu_{f}\left(t_{ref}-\beta t\right)}}$, where $B_{0}$ is a constant.
- The temperatures for the wall surface and far-field region are symbolized as $T_{w}$ and $T_{\infty }$, respectively.
- This model excludes the effect of sedimentation/aggregation since the hybrid nanofluid is assumed to be stably synthesized.

The USSP (unsteady separated stagnation point) flow with energy equations were presented in [28,43,44].

(1) $$\frac{\partial u\;}{\partial x}+\frac{\partial v}{\partial y}=0,$$

(2) $$\;\frac{\partial u}{\partial t}+u\frac{\partial u}{\partial x}+v\frac{\partial u}{\partial y}=\frac{\partial u_{e}}{\partial t}+u_{e}\frac{\partial u_{e}}{\partial x}+\frac{\mu_{hff}}{\rho_{hff}}\frac{\partial^{2}u}{\partial y^{2}}-\frac{\sigma_{hff}B_{1}^{2}}{\rho_{hff}}\left(u-u_{e}\right)$$

(3) $$\frac{\partial T}{\partial t}+u\frac{\partial T}{\partial x}+v\frac{\partial T}{\partial y}=\frac{k_{hff}}{{\left(\rho C_{p}\right)}_{hff}}\frac{\partial^{2}T}{\partial y^{2}}+\frac{Q_{1}}{{\left(\rho C_{p}\right)}_{hff}}\left(T-T_{\infty }\right),$$

(4) $$\begin{array}{l} u\left(x,y,t\right)=u_{0}\left(t\right),v\left(x,y,t\right)=0,T\left(x,y,t\right)=T_{w}\;\mathrm{at}\;y=0\\ u\left(x,y,t\right)\to u_{e}\left(x,t\right),T\left(x,y,t\right)\to T_{\infty }\;\mathrm{as}\;y\to \infty \end{array}\}.$$

In these equations, the hybrid nanofluid velocities are symbolized as u and v, and T is the temperature. Following Dholey [43] and Khashi’ie et al. [44], the appropriate transformation for Equations (2)–(4) which fulfills Equation (1) is as follows:

(5) $$u=\alpha \frac{x-x_{0}\left(t\right)}{t_{ref}-\beta t}f^{\prime }\left(\eta \right)+u_{0}\left(t\right),v=-\alpha \sqrt{\frac{\nu_{f}}{t_{ref}-\beta t}}f\left(\eta \right),\theta \left(\eta \right)=\frac{T-T_{\infty }}{T_{w}-T_{\infty }},\eta =\frac{y}{\sqrt{\nu_{f}\left(t_{ref}-\beta t\right)}}\}.$$

The following ordinary (similarity) differential equations can be obtained by substituting Equation (5) into Equations (2)–(4):

(6) $$\frac{\mu_{hff}/\mu_{f}}{\rho_{hff}/\rho_{f}}f\prime \prime \prime +\alpha \left(ff\prime \prime -f{’}^{2}+1\right)-\beta \left(\frac{1}{2}\eta f\prime \prime +f’-1\right)-\frac{\sigma_{hff}/\sigma_{f}}{\rho_{hff}/\rho_{f}}M^{2}\left(f’-1\right)=0,$$

(7) $$\frac{k_{hff}/k_{f}}{\mathrm{Pr}{\left(\rho C_{p}\right)}_{hff}/{\left(\rho C_{p}\right)}_{f}}\theta \prime \prime +\alpha f\theta ’-\frac{1}{2}\beta \eta \theta ’+\frac{Q}{{\left(\rho C_{p}\right)}_{hff}/{\left(\rho C_{p}\right)}_{f}}=0,$$

with the reduced BCs

(8) $$f\left(0\right)=0,f^{\prime }\left(0\right)=0,\theta \left(0\right)=1,f^{\prime }\left(\infty \right)\to 1,\theta \left(\infty \right)\to 0,$$

where $M^{2}=\sigma_{f}B_{0}^{2}/{\left(\nu \rho \right)}_{f}$ is the Hartmann number or magnetic parameter, $\mathrm{Pr}={\left(\mu C_{p}\right)}_{f}/k_{f}$ is the Prandtl number, and $Q=Q_{0}/{\left(\rho C_{p}\right)}_{f}$ is the heat generation parameter. The properties of water, magnetite, and cobalt ferrite for the computational analysis are listed in Table 1 [4]. Meanwhile, the correlations of the hybrid nanofluid properties which have been experimentally validated are shown in Table 2 [45].

According to Ao Roşca et al. [46] and Zainal et al. [47], the main interests of the physical quantities for USSP flow with heat transfer are the skin friction coefficient $f^{\prime \prime }\left(0\right)$ and heat transfer coefficient $-\theta^{\prime }\left(0\right)$. Following Roşca et al. [46], the streamline function $\bar{\psi }$ (for graphical purposes) is defined as

(9) $$x=\bar{\psi }=\frac{\psi }{\alpha }\frac{\sqrt{t_{ref}-\beta t}}{f\left(\eta \right)},$$

where

(10) $$\psi =\alpha \frac{x}{\sqrt{t_{ref}-\beta t}}f\left(\eta \right).$$

The availability of multiple solutions from Equations (6)–(8) is possible under the circumstance of unsteady decelerating flow. Hence, the effect of physical factors such as the accelerating parameter α, magnetic parameter M, and heat generation parameter Q in the production of the dual solutions is discussed in the Section 4. In general, for unsteady accelerating flow and steady flow, the values of $f^{\prime \prime }\left(0\right)$ are always positive, denoting the attached flow solution (AFS). However, $f^{\prime \prime }\left(0\right)$ can be either positive (AFS) or negative, which explains the reverse flow solution behavior (RFS).

## 3. Stability Analysis

Stability analysis is crucial in the determination of the real solution among other available solutions. Following early studies on stability analysis by Merkin [48], Weidman et al. [49], and Harris et al. [50], the following transformation is considered:

(11) $$\begin{array}{l} u=\alpha \frac{x-x_{0}\left(t\right)}{t_{ref}-\beta t}\frac{\partial f\left(\eta ,\tau \right)}{\partial \eta }+u_{0}\left(t\right),v=-\alpha \sqrt{\frac{\nu_{f}}{t_{ref}-\beta t}}f\left(\eta ,\tau \right),\theta \left(\eta ,\tau \right)=\frac{T-T_{\infty }}{T_{w}-T_{\infty }},\\ \eta =\frac{y}{\sqrt{\nu_{f}\left(t_{ref}-\beta t\right)}},\tau =\frac{\alpha t}{t_{ref}-\beta t}\;(\mathrm{time}\;\mathrm{variable}) \end{array}\}.$$

The following differential equations are obtained:

(12) $$\begin{array}{l} \frac{\mu_{hff}/\mu_{f}}{\rho_{hff}/\rho_{f}}\frac{\partial^{3}f}{\partial \eta^{3}}+\alpha \left(f\frac{\partial^{2}f}{\partial \eta^{2}}-{\left(\frac{\partial f}{\partial \eta }\right)}^{2}+1\right)-\beta \left(\frac{1}{2}\eta \frac{\partial^{2}f}{\partial \eta^{2}}+\frac{\partial f}{\partial \eta }-1\right)\\ -\frac{\sigma_{hff}/\sigma_{f}}{\rho_{hff}/\rho_{f}}M^{2}\left(\frac{\partial f}{\partial \eta }-1\right)-\alpha \left(1+\beta \tau \right)\frac{\partial^{2}f}{\partial \eta \partial \tau }=0, \end{array}$$

(13) $$\frac{k_{hff}/k_{f}}{\mathrm{Pr}{\left(\rho C_{p}\right)}_{hff}/{\left(\rho C_{p}\right)}_{f}}\frac{\partial^{2}\theta }{\partial \eta^{2}}+\alpha f\frac{\partial \theta }{\partial \eta }-\frac{1}{2}\beta \eta \frac{\partial \theta }{\partial \eta }+\frac{Q}{{\left(\rho C_{p}\right)}_{hff}/{\left(\rho C_{p}\right)}_{f}}\theta -\alpha \left(1+\beta \tau \right)\frac{\partial \theta }{\partial \tau }=0,$$

after the substitution of Equation (11) into Equations (2)–(4), while the transformed boundary conditions are

(14) $$f\left(0,\tau \right)=0,\;\frac{\partial f}{\partial \eta }\left(0,\tau \right)=0,\theta \left(0,\tau \right)=1,\;\frac{\partial f}{\partial \eta }\left(\infty ,\tau \right)\to 1,\;\theta \left(\infty ,\tau \right)\to 0.$$

The perturbation function is designed to test the possible disturbance in all the similarity solutions (see Weidman et al. [49]).

(15) $$\begin{array}{l} f\left(\eta ,\tau \right)=f_{0}\left(\eta \right)+e^{-\gamma \tau }F\left(\eta ,\tau \right),\\ \theta \left(\eta ,\tau \right)=\theta_{0}\left(\eta \right)+e^{-\gamma \tau }G\left(\eta ,\tau \right) \end{array}$$

where F,G are related to the similarity solution $f_{0},\theta_{0}$, while γ is the tested eigenvalue. The linearized eigenvalue equations are generated by substituting Equation (15) into Equations (12)–(14). The details of the procedure can be read in Weidman et al. [49] and Khashi’ie et al. [44]. The linearized equations are as follows:

(16) $$\frac{\mu_{hff}/\mu_{f}}{\rho_{hff}/\rho_{f}}F\prime \prime \prime +\alpha \left(f_{0}F\prime \prime +Ff_{0}\prime \prime -2f_{0}’F’\right)-\beta \left(\frac{1}{2}\eta F\prime \prime +F’\right)-\frac{\sigma_{hff}/\sigma_{f}}{\rho_{hff}/\rho_{f}}M^{2}F’+\alpha \gamma F’=0,$$

(17) $$\frac{k_{hff}/k_{f}}{\mathrm{Pr}{\left(\rho C_{p}\right)}_{hff}/{\left(\rho C_{p}\right)}_{f}}G\prime \prime +\alpha \left(f_{0}G’+F\theta_{0}’\right)-\frac{1}{2}\beta \eta G’+\left(\frac{Q}{{\left(\rho C_{p}\right)}_{hff}/{\left(\rho C_{p}\right)}_{f}}+\alpha \gamma \right)G=0,$$

(18) $$\begin{array}{l} F(0)=0,\;F^{\prime }(0)=0,\;F^{\prime \prime }(0)=0\;(\mathrm{replaced}),\;G(0)=0,\\ F^{\prime }(\eta )\to 0\;(\mathrm{relaxed})\;,G(\eta )\to 0\mathrm{as}\eta \to \infty . \end{array}$$

For successful generation of the smallest eigenvalues, the boundary condition ${F^{\prime }}_{0}(\eta )\to 0\mathrm{as}\eta \to \infty$ is substituted with a relaxing condition $F^{\prime \prime }(0)=1$, as mentioned by Harris et al. [45]. It is worth highlighting that the Equation (17) in this study can be reduced to Equation (17) in Khashi’ie et al.’s study [44] by letting Q=0. Hence, on the basis of the results of Khashi’ie et al. [44], it is justified that the first solution is real while the second solution is unstable under the case of Q=0.

## 4. Results and Discussion

**Figure 2:**
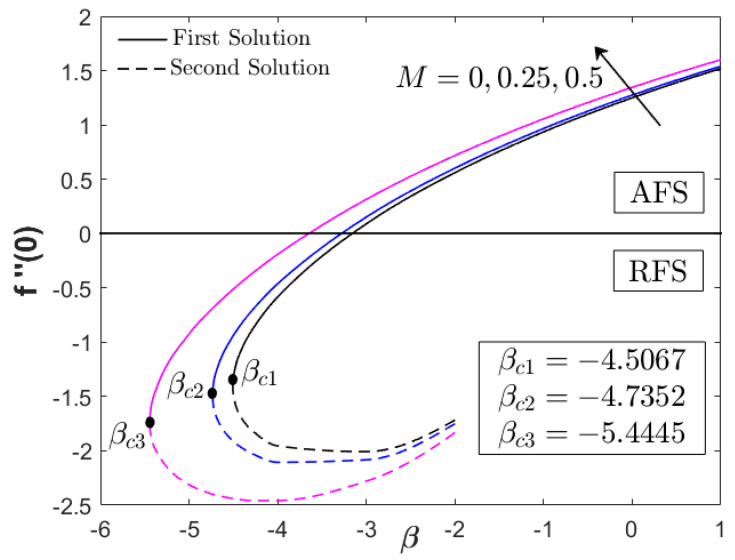
$f^{\prime \prime }\left(0\right)$ for various M (magnetic parameter).

**Figure 3:**
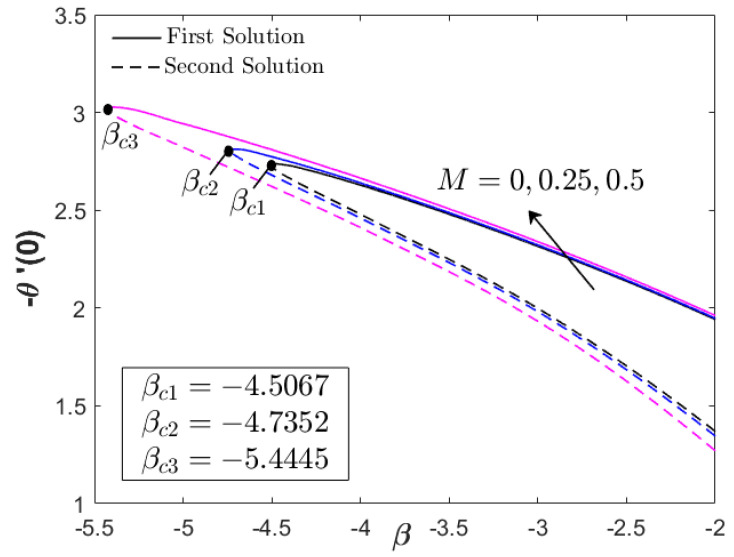
$-\theta^{\prime }\left(0\right)$ for various M (magnetic parameter).

**Figure 4:**
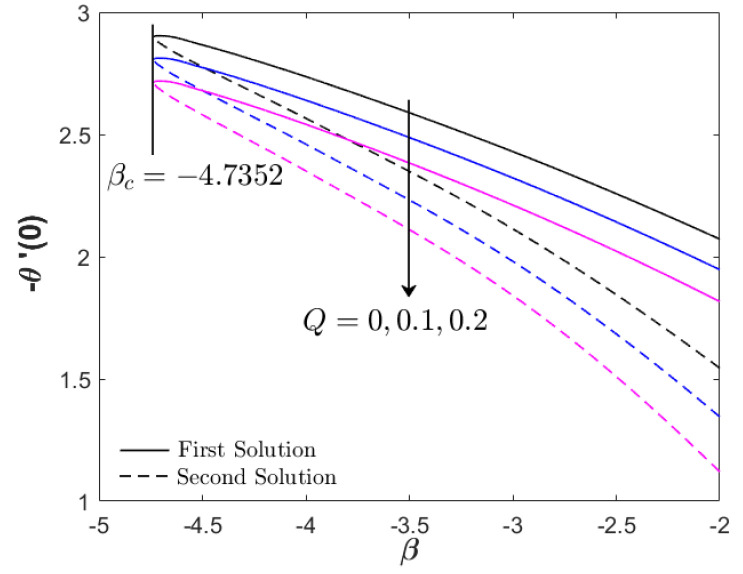
$-\theta^{\prime }\left(0\right)$ for various Q (heat generation parameter).

**Figure 5:**
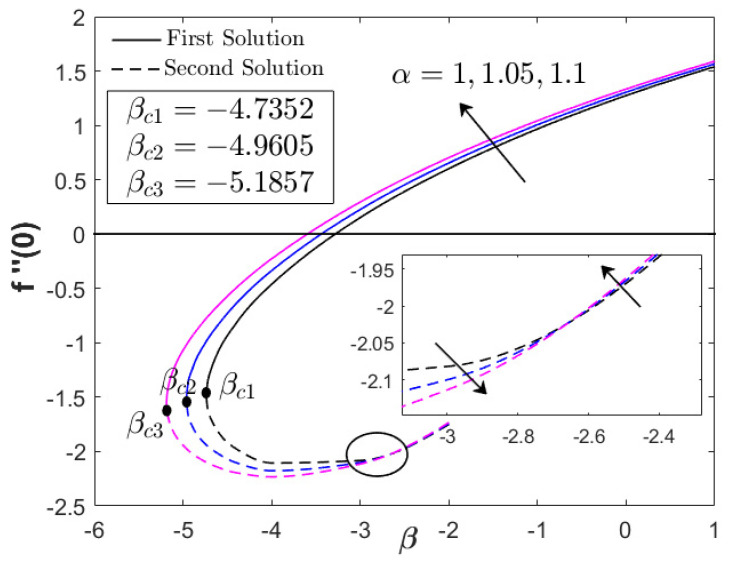
$f^{\prime \prime }\left(0\right)$ for various α (acceleration parameter).

**Figure 6:**
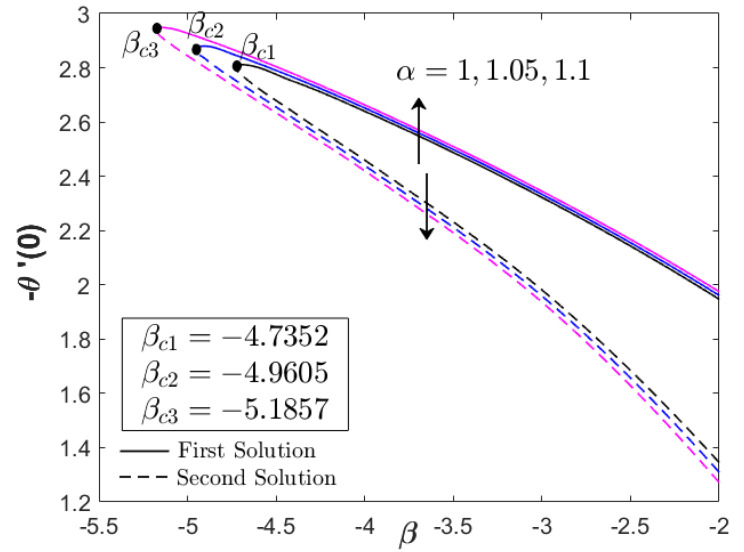
$-\theta^{\prime }\left(0\right)$ for various α (acceleration parameter).

**Figure 7:**
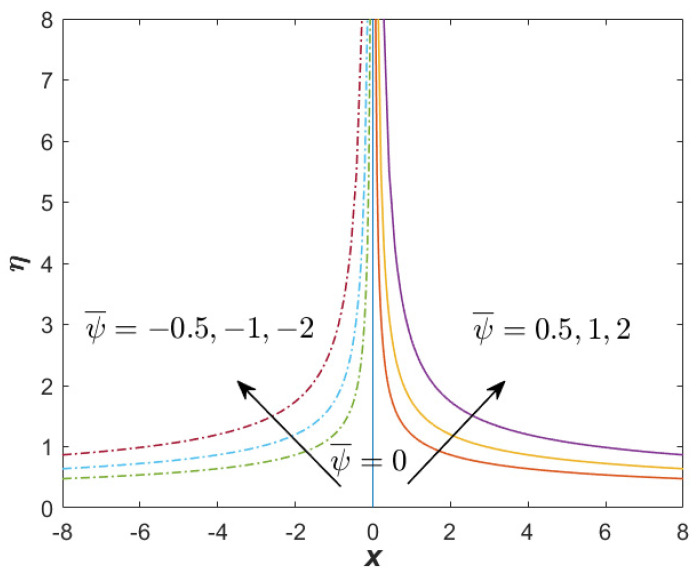
Streamline for the first (real) solution when α=1.1, β=−3 (unsteady decelerating flow), M=0.25, Q=0.1, and $\phi_{hff}=0.02$.

**Figure 8:**
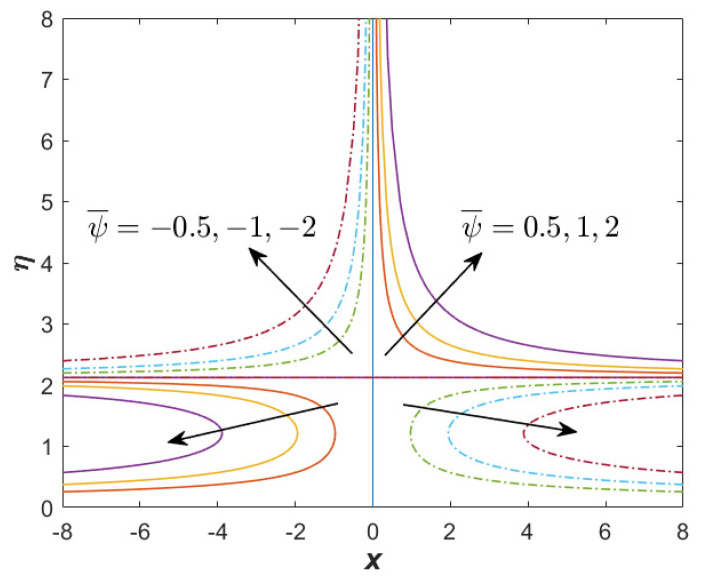
Streamline for the unique solution when α=1.1, β=−3 (unsteady accelerating flow), M=0.25, Q=0.1, and $\phi_{hff}=0.02$.

**Figure 9:**
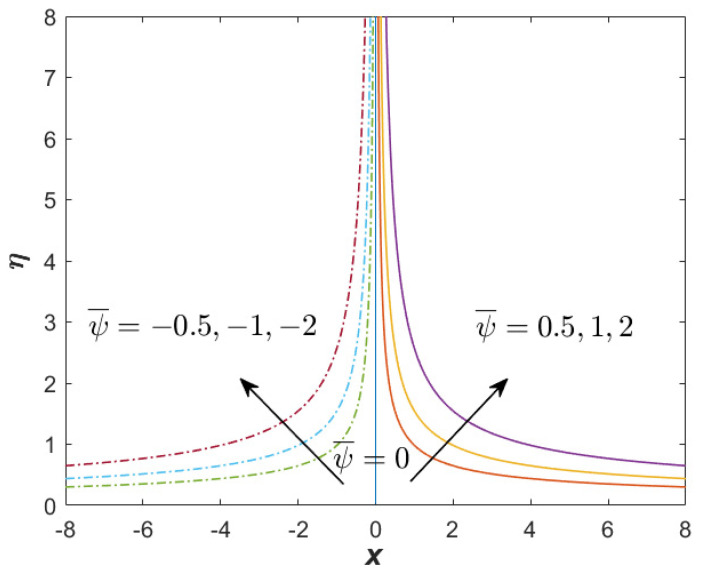
Streamline for the unique solution when α=1.1, β=1 (unsteady accelerating flow), M=0.25, Q=0.1, and $\phi_{hff}=0.02$.

**Figure 10:**
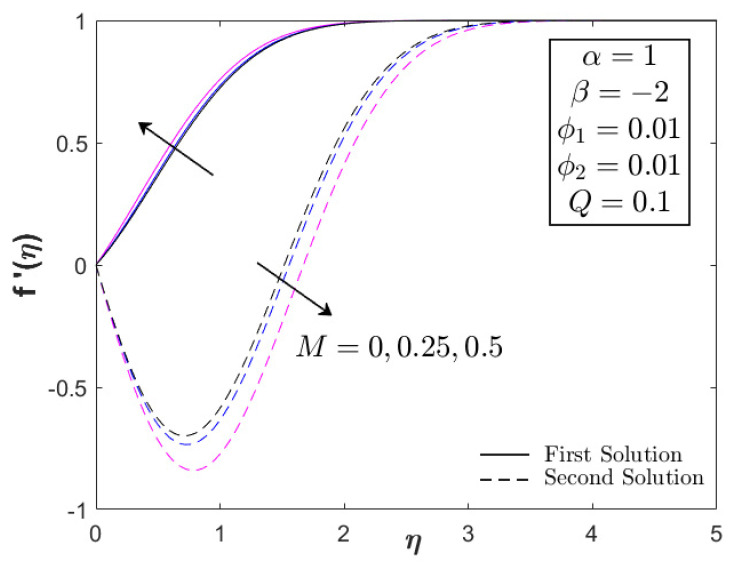
Velocity profile of Fe_3_O_4_–CoFe_2_O_4_/H_2_O with different M.

**Figure 11:**
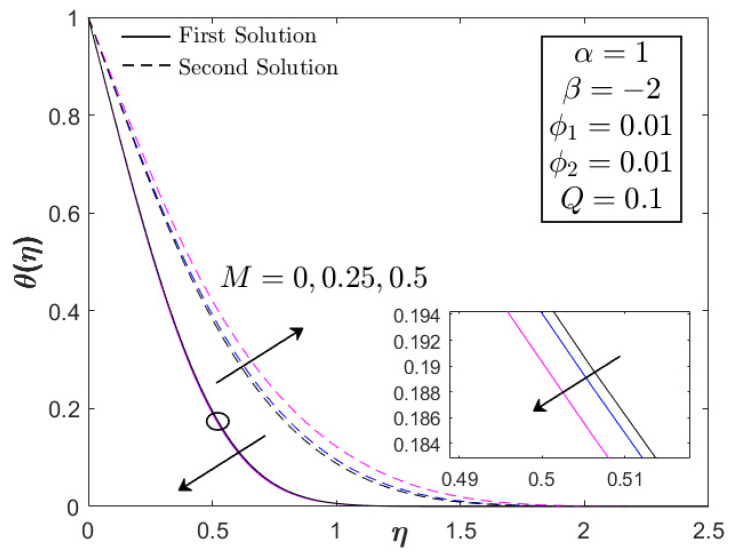
Temperature profile of Fe_3_O_4_–CoFe_2_O_4_/H_2_O with different M.

**Figure 12:**
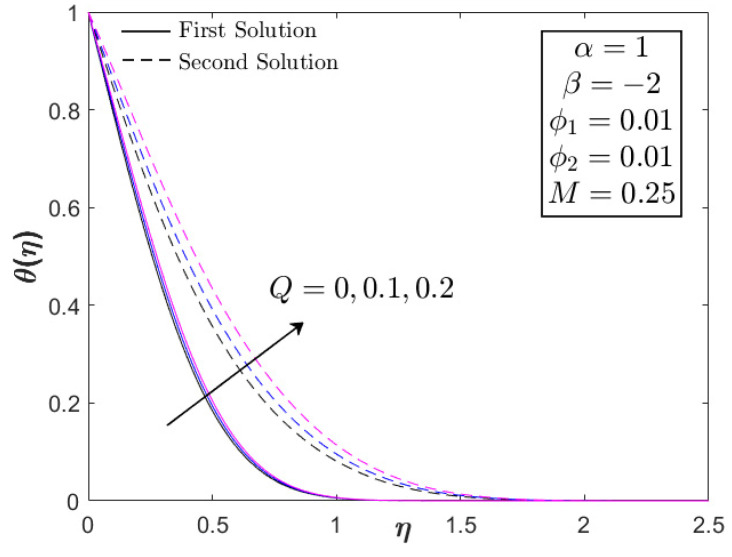
Temperature of Fe_3_O_4_–CoFe_2_O_4_/H_2_O with different Q.

This section provides the results obtained from the bvp4c (Matlab) solver by computing Equations (6)–(8) for the similarity solutions. The thermal and flow performances of Fe_3_O_4_–CoFe_2_O_4_/H_2_O were observed and analyzed, as displayed in Figure 2, Figure 3, Figure 4, Figure 5, Figure 6, Figure 7, Figure 8, Figure 9, Figure 10, Figure 11 and Figure 12 for variations of the magnetic parameter/MHD effect M, heat generation parameter Q, and acceleration parameter α subjected to Pr=6.2 (water), $0\leq \phi_{1},\phi_{2}\leq 0.01$, 1≤α≤1.1, 0≤M≤0.5, 0≤Q≤0.1, and $\beta_{c}\leq \beta \leq 1$. For the model’s accuracy and validity, a few solutions are validated by comparing them with the existing literature, as presented in Table 3 and Table 4. The approximate percentage relative errors $\left(\varepsilon_{a}\right)$ are also provided in the tables, as calculated using $\left(\varepsilon_{a}=\left|\frac{\mathrm{present}\;\mathrm{solution}-\mathrm{previous}\;\mathrm{solution}}{\mathrm{present}\;\mathrm{solution}}\right|\times 100\%\right)$. The approximate percentage relative errors between present and previous studies approach 0%, implying the accuracy of the solution. Meanwhile, Table 5 compiles the critical or separation values with different M, Q, and A, which are obtainable from Figure 2, Figure 3, Figure 4, Figure 5 and Figure 6. Furthermore, the critical values available in Khashi’ie et al. [44] when Cu–Al_2_O_3_/H_2_O is considered are assembled in Table 5. Without the heat generation factor and α=1 (usual stagnation point flow problem), it seems that the Fe_3_O_4_–CoFe_2_O_4_/H_2_O could extend the critical values beyond those of Cu–Al_2_O_3_/H_2_O with the upsurge of M. This shows the high capability of present hybrid nanofluid (Fe_3_O_4_–CoFe_2_O_4_/H_2_O) in delaying the boundary layer separation.

Figure 2, Figure 3 and Figure 4 display the impacts of M and Q on the progress of $f^{\prime \prime }\left(0\right)$ and $-\theta^{\prime }\left(0\right)$ toward the unsteadiness parameter $\beta_{c}\leq \beta \leq 1$. Dual solutions are detected within specific use of the physical factors and are available up to a separation/critical value $\beta_{c}$. The non-uniqueness of the solutions is possible due to the thickening of the boundary layer from the unstable vortices within boundary layer flow [43]. The vortices usually appear in the cases with unsteady decelerating and shrinking flow, which can be stabilized with the help of stagnation point flow. As stated in the previous section, the values of $f^{\prime \prime }\left(0\right)$ can be positive (known as AFS, attached flow solution) or negative (RFS, reverse flow solution) when β<0 is considered. A few papers also considered $\beta_{FS}$ or the separation value from AFS to RFS [43,44]. However, the main concern in the present paper is $\beta_{c}$ which separates the laminar and turbulent flows, as depicted in Figure 2, Figure 3 and Figure 4. It is apparent that the inclusion of the magnetic parameter can expand $\beta_{c}$, whereby $\beta_{c1}=-4.5067,$ $\beta_{c2}=-4.7352$, and $\beta_{c3}=-5.4445$ when M=0,0.25,0.5, highlighting that the magnetic field is a good factor in delaying the boundary layer separation process. All critical values were in the RFS region. Furthermore, the magnetic parameter increased both $f^{\prime \prime }\left(0\right)$ and $-\theta^{\prime }\left(0\right)$ for all values of β. However, as $\beta \to \beta_{c},$ a reduction in $f^{\prime \prime }\left(0\right)$ and an upsurge of $-\theta^{\prime }\left(0\right)$ could be observed. The increase in magnetic parameter physically develops the Lorentz force, which opposes the boundary layer flow. However, the USSP behavior in the Fe_3_O_4_–CoFe_2_O_4_/H_2_O flow helps in stabilizing the detached vorticity, which accelerates the skin friction coefficient, as well as the fluid motion, as portrayed in Figure 2 and Figure 10. Meanwhile, as the fluid velocity is enhanced, the hot particles within the fluid are driven and transferred into the cool plate, revealing the active operation of thermal transfer (see Figure 3).

As shown in Figure 4, the increment in heat generation parameter did not directly affect the boundary layer separation, since $\beta_{c}=-4.7352$ remained for all values of Q. The augmentation of heat generation parameter did not physically influence the distribution of the skin friction coefficient or the velocity profile. However, the addition of the heat generation parameter up to 10% reduced the heat transfer progress of Fe_3_O_4_–CoFe_2_O_4_/H_2_O as displayed in Figure 4. This highlights the effectiveness of heat absorption as compared to the heat generation in the thermal flow process. However, as $\beta \to \beta_{c},$ an increment in $-\theta^{\prime }\left(0\right)$ was discovered. Figure 5 and Figure 6 illustrate the effect of the acceleration parameter α on $f^{\prime \prime }\left(0\right)$ and $-\theta^{\prime }\left(0\right)$, respectively. It is apparent that both distributions increased as a function of α. Physically, the acceleration parameter signifies the strength of the stagnation point flow in preserving the unconfined vorticity. The accession of α induces the Fe_3_O_4_–CoFe_2_O_4_/H_2_O motion by intensifying the skin friction, as well as the thermal progress. As previously discussed in Figure 3, the progressive Fe_3_O_4_–CoFe_2_O_4_/H_2_O motion allocates the hot fluid particles into the cool plate, highlighting the active thermal progress, as shown in Figure 6.

Figure 7 and Figure 8 present the streamline function for the first and second solutions when α=1.1, β=−3 (unsteady decelerating flow), M=0.25, Q=0.1, and $\phi_{hff}=0.02$. The results generation of both solutions from the bvp4c solver show that the mesh grid was 139 (first) and 150 (second). This shows that the boundary layer thickness of the second solution was considerably greater than that of the first solution. Figure 7 proves that no reverse flow was produced for the first solution, while the reverse flow could be spotted in Figure 8 for the second solution. In Figure 8, there exists a symmetric streamline (stagnation line), which was not producible in the case of oblique stagnation point flow. The stagnation line for the second solution was greater than the first solution, which reflects the great thickness of the boundary layer for the second solution. Meanwhile, Figure 9 reflects the streamline function for the only solution when the case of β=1 (unsteadiness accelerating flow) is considered. Similar to Figure 7, no reverse flow is obtainable.

**Figure 13:**
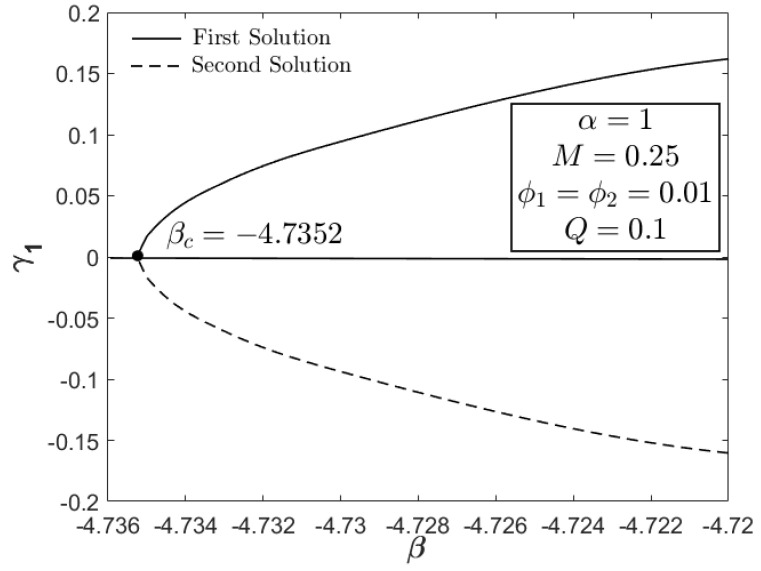
Analysis of solution stability.

Figure 10, Figure 11 and Figure 12 portray the profiles of velocity and temperature with different values of M and Q as the testing factors. All profiles fulfill the boundary conditions in Equation (8), indicating the legitimacy of the model. Furthermore, the velocity distribution (first solution) in Figure 10 expands with the increment in M, whereas the temperature distribution shows an adverse result, decreasing as a function of M. As stated before, the combination of magnetic field and USSP flow would not reduce the velocity profile since the presence of the acceleration parameter can stabilize the vorticity, thereby assisting the motion of Fe_3_O_4_–CoFe_2_O_4_/H_2_O. This is the physical reason for the increasing behavior of velocity in Figure 10. The reduction in temperature (first/real solution) in Figure 11 corresponds to the active process of the hot particle being transferred into the cool ambient surface. As discussed earlier, the heat generation parameter is not the contributing factor in the flow progress; hence, only the temperature profile is supplied in Figure 12. Both first and second solutions augment with the addition of Q, showing a reduction in the heat transfer progress. Figure 13 displays the stability analysis results from Equations (16)–(18), highlighting the reliability of the first solution with the smallest positive eigenvalues as $\beta \to \beta_{c}$. The accuracy of stability formulation could also validated from the trend of $\gamma_{1}\to 0$ as $\beta \to \beta_{c}$.

## 5. Conclusions

The unsteady separated stagnation point (USSP) flow consisting of two different nanoparticles (Fe_3_O_4_ (magnetite) and CoFe_2_O_4_ (cobalt ferrite)) was established with water as the working fluid. The flow was subjected to heat generation and magnetic field conditions. The conclusions from this study are as follows:

- The magnitude of the skin friction and the heat transfer coefficients is increased for larger magnetic and acceleration parameters.
- The heat transfer performance decreases with the imposition of heat generation.
- Larger values of the magnetic and acceleration parameters contribute to an expansion of the domain of the solutions, where they are terminated at certain points of the unsteadiness deceleration parameter.
- The heat generation parameter is not a developing factor in the flow and thermal progress of Fe_3_O_4_–CoFe_2_O_4_/H_2_O for the USSP flow case.
- Streamlines were presented to show the flow pattern, whereby the second solutions were split into two regions, while the first solution presented normal stagnation point flow.

## Figures and Tables

**Table 1:** Physical properties.

Properties	Water	Magnetite	Cobalt Ferrite
ρ $\;(\mathrm{kg}/m^{3})$	997.1	5180	4908
$C_{p}$ ( J/kg·K)	4179	670	700
k (W/m·K)	0.613	9.8	3.6
σ (S/m)	0.05	0.74 × 10^6^	1.1 × 10^7^
Prandtl number (Pr)	6.2	-	-

**Table 2:** General correlations of hybrid nanofluids.

Properties	Correlations
Thermal conductivity	$k_{hff}=\left[\frac{\left(\frac{\phi_{1}k_{1}+\phi_{2}k_{2}}{\phi_{hff}}\right)-2\phi_{hff}k_{f}+2\left(\phi_{1}k_{1}+\phi_{2}k_{2}\right)+2k_{f}}{\left(\frac{\phi_{1}k_{1}+\phi_{2}k_{2}}{\phi_{hff}}\right)+\phi_{hff}k_{f}-\left(\phi_{1}k_{1}+\phi_{2}k_{2}\right)+2k_{f}}\right]k_{f}$
Electrical conductivity	$\sigma_{hff}=\left[\frac{\left(\frac{\phi_{1}\sigma_{1}+\phi_{2}\sigma_{2}}{\phi_{hff}}\right)-2\phi_{hff}\sigma_{f}+2\left(\phi_{1}\sigma_{1}+\phi_{2}\sigma_{2}\right)+2\sigma_{f}}{\left(\frac{\phi_{1}\sigma_{1}+\phi_{2}\sigma_{2}}{\phi_{hff}}\right)+\phi_{hff}\sigma_{f}-\left(\phi_{1}\sigma_{1}+\phi_{2}\sigma_{2}\right)+2\sigma_{f}}\right]\sigma_{f}$
Heat capacity	${\left(\rho C_{p}\right)}_{hff}=\phi_{1}{\left(\rho C_{p}\right)}_{s1}+\phi_{2}{\left(\rho C_{p}\right)}_{s2}+\left(1-\phi_{hff}\right){\left(\rho C_{p}\right)}_{f}$
Density	$\rho_{hff}=\phi_{1}\rho_{s1}+\phi_{2}\rho_{s2}+\left(1-\phi_{hff}\right)\rho_{f}$
Dynamic viscosity	$\mu_{hff}=\frac{\mu_{f}}{{\left(1-\phi_{hff}\right)}^{2.5}};\phi_{hff}=\phi_{1}+\phi_{2}$

**Table 3:** Validation of $f^{\prime \prime }\left(0\right)$ when $\phi_{1}=\phi_{2}=Q=0,$ β=−1, and α=1, with various M.

M	First Solution			Second Solution		
	Present Study	[44]	[43]	Present Study	[44]	[43]
0	0.923204	0.923204 (0%)	0.9232 (0.0004%)	−0.985139	−0.985134 (0.0005%)	−0.9851 (0.0005%)
0.25	0.954505	0.954505 (0%)	0.9545 (0.0005%)	−0.961278	−0.961278 (0%)	−0.9613 (0.0023%)
0.50	1.043478	1.043478 (0%)	1.0435 (0.0021%)	−0.851369	−0.851369 (0%)	−0.8514 (0.0036%)
0.88	1.262495	1.262495 (0%)	1.2625 (0.0004%)	0.038797	0.038797 (0%)	0.0388 (0.0077%)
1	1.346660	1.346660 (0%)	1.3467 (0.0030%)	0.499050	0.499050 (0%)	0.4991 (0.0100%)

(%) Approximate percentage relative error.

**Table 4:** Validation of $-\theta^{\prime }\left(0\right)$ when $\phi_{1}=\phi_{2}=Q=0,$ β=−1, and α=1, with various M.

M	First Solution			Second Solution		
	Present Study	[44]	[43]	Present Study	[44]	[43]
0	1.675545	1.675545 (0%)	-	0.502540	0.502540 (0%)	-
0.25	1.680398	1.680398 (0%)	-	0.425526	0.425525 (0.0002%)	-
0.50	1.693818	1.693818 (0%)	-	0.188880	0.188880 (0%)	-
0.88	1.724685	1.724685(0%)	-	0.010937	0.010937 (0%)	-
1	1.735799	1.735799 (0%)	-	0.206980	0.206980 (0%)	-

(%) Approximate percentage relative error.

**Table 5:** Comparison of $\beta_{c}$ with Khashi’ie et al. [44] when $\phi_{1}=\phi_{2}=0.01$, with various parameters.

M	Q	α	$\beta_{c}(\mathrm{Present}\;\mathrm{Study},$ Fe_3_O_4_–CoFe_2_O_4_/H_2_O)	$\beta_{c}(\mathrm{Khashi}’\mathrm{ie}\;\mathrm{et}\;\mathrm{al}.$ [44], Cu–Al_2_O_3_/H_2_O)
0	0	1	−4.5067	−4.5066
0.25			−4.7352	−4.7294
0.5			−5.4445	−5.4199
		1.05	-	−5.6437
		1.10	-	−5.8677
0.25	0.1	1	−4.7352	-
	0.2		−4.7352	-
0.25	0.1	1.05	−4.9605	-
		1.1	−5.1857	-

## Data Availability

Not applicable.
